# Peripheral retinal degenerations: multimodal imaging–guided risk stratification and clinical decision-making

**DOI:** 10.3389/fopht.2026.1814997

**Published:** 2026-05-13

**Authors:** Siniša Babović, Nemanja Maletin, Nikola Denda

**Affiliations:** 1 Eye Clinic, University Clinical Center of Vojvodina, University of Novi Sad, Novi Sad, Serbia; 2 University of Novi Sad, Faculty of Medicine, Novi Sad, Serbia

**Keywords:** AI-assisted risk stratification, laser photocoagulation, lattice degeneration, PRD, PVD, RRD, RT, SS-OCT

## Abstract

**Introduction:**

Peripheral retinal degenerations (PRD) encompass a broad spectrum of peripheral retinal alterations, ranging from benign atrophic changes to tractional lesions associated with retinal tears (RT) and rhegmatogenous retinal detachment (RRD). Despite increasing detection with modern peripheral imaging, the clinical relevance and management of many PRD remain controversial. This review aims to provide an imaging guided, risk based framework for PRD evaluation and management, emphasizing the role of posterior vitreous detachment (PVD), selective prophylactic intervention, and emerging AI-assisted risk stratification and decision support concepts.

**Methods:**

A narrative literature search was conducted in PubMed, Scopus, and Web of Science (January 2000 to September 2025) using predefined terms related to PRD, vitreoretinal traction, RT, RRD, PVD, ultra widefield (UWF) imaging, and peripheral OCT. Of 842 records identified, 29 studies met inclusion criteria and were qualitatively synthesized.

**Results:**

PRD can be functionally stratified into low risk atrophic lesions (paving stone degeneration, WWOP), intermediate entities (degenerative retinoschisis), and high risk tractional lesions (lattice degeneration, snail track degeneration, retinal tufts). PVD represents the key biomechanical event linking PRD with RT and RRD, particularly during acute symptomatic stages. UWF imaging and SS-OCT improve visualization of peripheral morphology and vitreoretinal interface abnormalities, enabling differentiation between benign lesions, tractionally unstable configurations, and subclinical RRD. Current evidence supports selective prophylactic laser photocoagulation in eyes with symptomatic RT, fellow eye RRD, or OCT confirmed tractional instability, while routine treatment of asymptomatic low risk PRD is not justified. AI-assisted decision support may help standardize risk interpretation by integrating multimodal imaging features with clinical risk modifiers within a human in the loop model.

**Conclusion:**

PRD management should be individualized using integrated clinical assessment and multimodal imaging, with intervention targeted to tractional instability and meaningful risk modifiers.

## 1 Introduction

Peripheral retinal degenerations (PRD) represent a heterogeneous group of structural alterations affecting the peripheral retina that arise from a complex interplay of anatomical, metabolic, and vitreoretinal factors. The majority of these changes are detected incidentally during routine ophthalmic examinations and typically follow a benign clinical course. However, certain forms-particularly those associated with vitreoretinal traction-carry a clearly defined risk for the development of peripheral retinal tears (RT) and, under specific circumstances, rhegmatogenous retinal detachment (RRD).

Epidemiological data indicate that lattice degeneration occurs in approximately 8% of the general population, while it is present in up to 40% of eyes with RRD, underscoring its clinical importance and pathophysiological relevance in the context of retinal tear and detachment development (1, 2). The central mechanism linking PRD to the occurrence of RT and RRD is posterior vitreous detachment (PVD). With physiological aging of the vitreous body, a gradual process of liquefaction and separation from the inner limiting membrane of the retina takes place, resulting in localized tractional forces, particularly at sites of strong vitreoretinal adhesion. These areas often spatially coincide with degenerative retinal changes. When traction is acute, asymmetric, or focally pronounced, it may lead to the formation of a horseshoe-shaped retinal tear, most commonly in association with lattice or snail-track degeneration (3, 4).

The development of modern peripheral retinal imaging modalities has led to significant advances in the morphological assessment of the retinal periphery. Ultra-widefield (UWF) fundus imaging, peripheral optical coherence tomography (OCT), and OCT angiography (OCTA) enable detailed detection of subtle morphological alterations, elevated lesions, and tractional dynamics that are not always apparent on standard indirect ophthalmoscopy. Nevertheless, increased diagnostic sensitivity does not equate to increased clinical relevance-many PRD identified using contemporary imaging techniques have no realistic potential to progress to retinal tear or detachment and therefore do not require therapeutic intervention (5, 6).

The contemporary challenge in retinology therefore extends beyond detection of peripheral retinal lesions and increasingly centers on standardized interpretation and reliable differentiation between lesions that are biomechanically unstable and those with a stable, benign course. Degenerations such as paving-stone atrophy or WWOP typically require observation alone, whereas traction-associated lesions may warrant an active approach, including prophylactic laser photocoagulation in carefully selected scenarios. A clear understanding of the natural history of PRD, vitreoretinal biomechanics, and potential complications remains essential for appropriate clinical decision-making in everyday practice.

However, a relevant knowledge gap persists. Although modern peripheral imaging modalities such as UWF and SS-OCT enable increasingly detailed characterization of PRD and the vitreoretinal interface, there is still limited consensus on how specific imaging features should be translated into reproducible, risk-based management decisions. In particular, clinically oriented frameworks that integrate symptoms, PVD stage, refractive risk, and multimodal imaging signs of tractional instability into a standardized decision pathway remain scarce. As a result, prophylactic treatment thresholds vary substantially across clinicians and centers, highlighting the need for an imaging-guided, risk-stratified approach and for emerging decision support concepts, including AI-assisted risk stratification within a human-in-the-loop model, to improve consistency while minimizing unnecessary interventions.

## 2 Methodology

This study was conducted as a narrative review aimed at integrating current knowledge on peripheral retinal degenerations (PRD) and their clinical significance. A comprehensive literature search was performed in PubMed, Scopus, and Web of Science, covering the period from January 2000 to September 10, 2025. Combinations of controlled vocabulary terms and keywords related to peripheral retinal degenerations, vitreoretinal traction, posterior vitreous detachment (PVD), retinal tears, rhegmatogenous retinal detachment (RRD), and contemporary diagnostic modalities, including UWF imaging, peripheral OCT, and swept-source OCT, were used.

**Figure 1:**
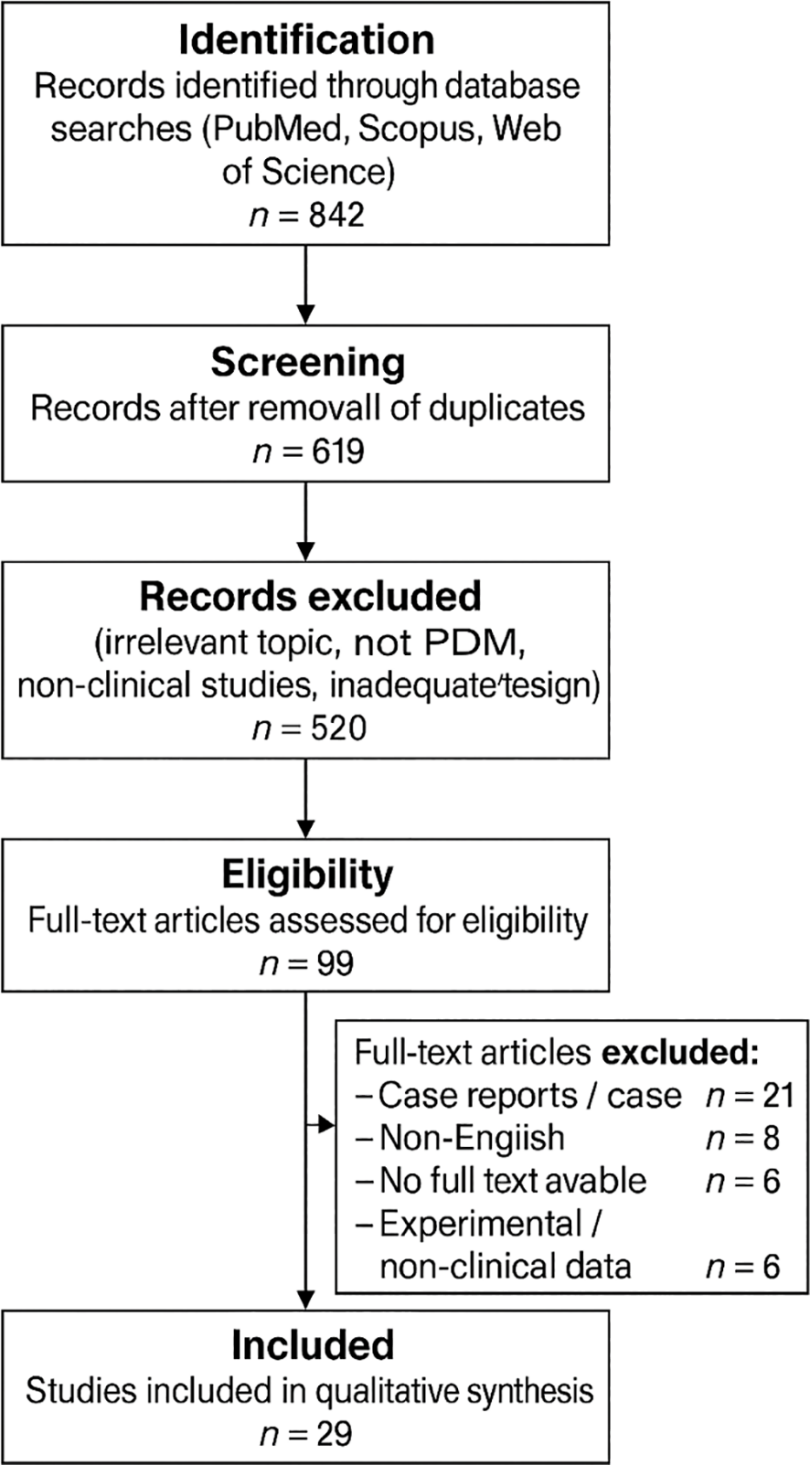
PRISMA flow diagram of literature selection process.

In addition to the electronic database search, the reference lists of relevant articles were manually screened to identify additional eligible studies. The review considered prospective and retrospective clinical studies, cohort and observational studies, as well as systematic reviews and meta-analyses addressing the epidemiology, morphology, natural history, biomechanics of the vitreoretinal interface, and diagnostic evaluation of PRD. Studies were selected according to predefined inclusion and exclusion criteria (**Figure 1**).

The initial search identified 842 records. After duplicate removal, two investigators independently screened titles, abstracts, and subsequently full-text articles for eligibility. Any disagreements regarding study selection, eligibility assessment, or interpretation of the findings were resolved through discussion and consensus; when consensus could not be reached, the senior author served as an adjudicator. Although this was a narrative review, we incorporated PRISMA-informed elements to improve methodological transparency and reproducibility. Because of substantial methodological heterogeneity among the included studies, a quantitative meta-analysis was not considered appropriate. Therefore, a qualitative narrative synthesis was performed, with emphasis on shared pathophysiological mechanisms, differences in risk profiles across PRD subtypes, and the clinical implications of multimodal diagnostic evaluation. In interpreting the findings, particular attention was given to major potential sources of bias, including the predominance of retrospective study designs, possible selection bias, inconsistent definitions of PRD, variability in imaging protocols and modality use (UWF, SD-OCT, SS-OCT), and nonuniform indications and thresholds for prophylactic laser treatment across studies.

### 2.1 Pathophysiology of peripheral retinal degenerations

The peripheral retina represents an anatomically and functionally critical zone for overall retinal stability. Its close relationship with the vitreous body and the ora serrata provides mechanical support and contributes to the structural integrity of the retinal periphery. Strong vitreoretinal adhesions act as stabilizing anchors, particularly in younger individuals with a homogeneous vitreous firmly attached to the inner limiting membrane (7, 8). With ocular aging and in myopic eyes, these adhesions progressively weaken, while vitreous liquefaction and loss of cohesiveness promote the formation of abnormal focal adhesions at the retinal periphery, increasing susceptibility to posterior vitreous detachment (PVD) and tractional retinal tears (9, 10).

The peripheral retina also exhibits distinct metabolic and hemodynamic characteristics. Compared with the macula, it has relatively lower perfusion but remains metabolically active, particularly in oxidative stress regulation and nutrient diffusion. Degenerative changes, whether atrophic or tractional, may disrupt this balance and predispose the retina to further structural compromise (8, 11).

**Figure 2:**
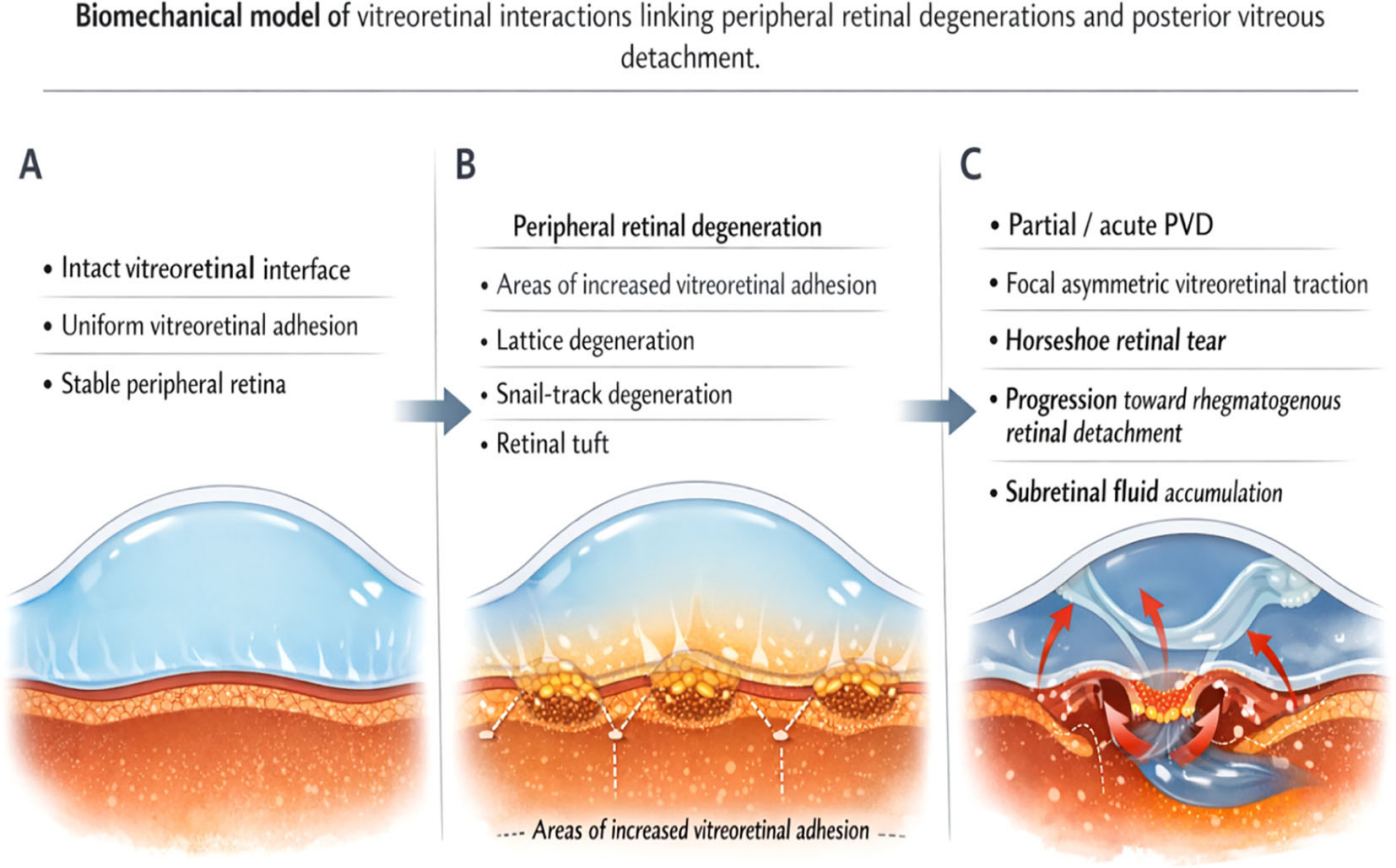
Biomechanical model of vitreoretinal interactions linking peripheral retinal degenerations and posterior vitreous detachment. **(A)** Stable peripheral retina with an intact vitreoretinal interface and uniform vitreoretinal adhesion. **(B)** Peripheral retinal degeneration with areas of increased vitreoretinal adhesion, including lattice degeneration, snail-track degeneration, and retinal tuft. **(C)** Partial/acute posterior vitreous detachment with focal asymmetric vitreoretinal traction, horseshoe retinal tear formation, progression toward rhegmatogenous retinal detachment, and subretinal fluid accumulation. The schematic illustrates how peripheral retinal degenerations with increased vitreoretinal adhesion predispose to focal traction during partial or acute posterior vitreous detachment, leading to horseshoe retinal tear formation and potential progression toward rhegmatogenous retinal detachment.

PVD represents the central pathophysiological event linking peripheral retinal degenerations with retinal tear formation. Age-related vitreous syneresis, collagen network collapse, and loss of hyaluronic acid–mediated stability render the peripheral retina thinner and more vulnerable at the onset of vitreous separation (9, 10). During acute or incomplete PVD, asymmetric vitreoretinal traction may lead to horseshoe-shaped retinal tears, most commonly in association with lattice and snail-track degeneration (12, 13). Additional factors, including cataract surgery, laser capsulotomy, refractive procedures, or intravitreal injections, may accelerate the development of secondary, iatrogenically induced PVD and further increase the risk of retinal tears and rhegmatogenous retinal detachment (9, 10). The biomechanical relationship between peripheral retinal degenerations and PVD is illustrated in **Figure 2**.

In myopic eyes, axial elongation further accentuates peripheral retinal thinning and alters vitreoretinal biomechanics, increasing the prevalence of tractional lesions and their propensity for tear formation (8, 11). Reduced perfusion may also contribute to peripheral retinal atrophy and the development of lesions such as paving-stone degeneration, further compromising local biomechanical stability and increasing susceptibility to vitreoretinal traction (14). Across clinically significant peripheral retinal degenerations, the shared pathogenic features include retinal thinning, abnormal vitreoretinal adhesion, and dynamic traction during PVD, explaining their role as preferential sites for retinal break development (12, 13).

### 2.2 Classification of peripheral retinal degenerations

Peripheral retinal degenerations encompass a broad spectrum of structural changes affecting the equatorial and extreme peripheral retina, with their clinical significance largely determined by lesion morphology, the presence of vitreoretinal traction, and the propensity for progression to peripheral retinal tears and rhegmatogenous retinal detachment. Contemporary literature emphasizes that a functional, risk-based classification provides the most precise framework for clinical decision-making, particularly when combined with modern UWF imaging and SS-OCT, which reveal peripheral retinal details that were previously undetectable (14–16).

Low-risk lesions include changes such as paving-stone atrophy, white-without-pressure (WWOP), and microretinal degenerations, which do not carry an inherent risk for retinal tear development and almost never require intervention. Recent UWF and SS-OCT studies confirm that, despite their sometimes pronounced morphological appearance, these lesions do not exhibit features of vitreoretinal traction or dynamic changes suggestive of tear formation (14, 16). Paving-stone atrophy represents localized areas of retinal pigment epithelium (RPE) and choroidal atrophy associated with reduced choroidal perfusion, whereas WWOP predominantly reflects an optical phenomenon and subtle alterations in vitreoretinal contact, without an increased risk even in eyes with high refractive error (16, 17).

Moderate-risk lesions, among which degenerative retinoschisis occupies a distinct position, generally demonstrate a stable natural course. However, in the presence of high axial myopia, acute PVD, or a history of retinal detachment in the fellow eye, these lesions may acquire clinical relevance. Recent SS-OCT–based studies indicate that retinoschisis is often morphologically more complex than previously assumed, with clearly delineated inner and outer retinal layers and possible microcommunications that are better visualized using UWF imaging (14, 15). A significant increase in risk occurs primarily when breaks are present in both retinal layers, allowing direct communication between the vitreous cavity and the subretinal space, thereby facilitating progression toward RRD (15).

High-risk lesions are of greatest clinical importance, with lattice degeneration remaining the most significant entity. Contemporary SS-OCT investigations confirm that lattice degeneration is characterized by pronounced retinal thinning, fragmentation of the Henle fiber layer, microtractional adhesions, and clearly defined vitreoretinal “bridging” structures that were not previously visualizable (14). Epidemiological data remain consistent with earlier reports: lattice degeneration occurs in approximately 8% of the general population but is identified in a substantial proportion of eyes with rhegmatogenous retinal detachment, a finding corroborated by modern UWF-based population studies (16, 17). Studies published between 2023 and 2025 emphasize that the posterior margin of lattice lesions is particularly susceptible to tractional horseshoe tear formation during acute PVD, representing the principal mechanism leading to RRD (14, 18).

Snail-track degeneration, previously considered a variant of lattice degeneration, is increasingly recognized in contemporary literature as a distinct clinical entity. The most recent classification (19) describes snail-track degeneration as a linear, hyporeflective zone with microgranular reflective surfaces and markedly thinned outer retinal layers, rendering it highly susceptible to tractional forces. In the setting of acute PVD, these lesions demonstrate a risk of retinal tear formation comparable to that of lattice degeneration.

Retinal tufts, particularly meridional and nonpigmented forms, have been confirmed by modern UWF and SS-OCT imaging as focal sites of abnormally strong vitreoretinal adhesion (19). These lesions represent frequent points of initial tractional tear formation during PVD, with a higher prevalence observed in young myopic individuals and in eyes with an extended vitreoretinal base (17). Their clinical relevance is especially pronounced in acute symptomatic presentations, where their presence significantly increases the likelihood of identifying a fresh retinal tear.

Ultimately, the functional classification of peripheral retinal degenerations is not merely an academic construct but a cornerstone of contemporary clinical practice. Recent reviews from 2024–2025 emphasize that classification should not be applied in isolation but rather interpreted within the broader context of vitreoretinal status, refractive profile, presence or absence of PVD, and planned ophthalmic procedures (17, 18). This integrated approach enables individualized management strategies, ranging from observation alone to prophylactic laser photocoagulation in selected high-risk scenarios.

### 2.3 Diagnostic evaluation of the retinal periphery

Diagnostic assessment of the retinal periphery represents one of the most demanding aspects of clinical retinology, as many peripheral retinal degenerations present subtly, without obvious elevation or symptoms, while some carry a substantial risk for the development of peripheral retinal tears and rhegmatogenous retinal detachment. Indirect ophthalmoscopy with scleral depression remains the cornerstone of peripheral retinal examination and continues to be regarded as the gold standard for evaluating the vitreoretinal interface, sites of abnormally firm vitreoretinal adhesion, and the presence of subretinal fluid. Multimodal imaging may provide valuable complementary information for documentation, lesion characterization, and follow-up, but it should not be regarded as a substitute for clinical examination. Despite ongoing technological advances, scleral depression is the only technique that enables real-time, three-dimensional assessment of the peripheral retina and remains indispensable for evaluating tractional dynamics, particularly in the setting of PVD.

In recent years, however, substantial progress has been achieved in peripheral retinal visualization, primarily owing to the development of UWF imaging. UWF systems, especially those based on scanning laser ophthalmoscopy (SLO), allow visualization of up to 200° of the peripheral retina in a single image, significantly increasing the detection rate of lattice degeneration, snail-track lesions, and retinal tufts. Contemporary UWF studies have demonstrated that these lesions are identified more reliably than with conventional fundus photography and that UWF imaging enables more consistent longitudinal follow-up (20, 21). UWF imaging is particularly useful for documenting morphologic changes that evolve over time and for identifying multiple peripheral lesions that may be difficult to detect during routine examination without scleral depression.

Another major diagnostic advance is the application of SS-OCT in the analysis of the peripheral retina. Compared with spectral-domain OCT (SD-OCT), SS-OCT provides deeper tissue penetration and a more stable signal in peripheral regions, allowing visualization of retinal layers far beyond the macular area. Recent studies have described detailed microstructural features of lattice degeneration using SS-OCT, including thinning of the outer retinal layers, fragmentation of photoreceptor segments, subclinical microhemorrhagic points, and bridging vitreoretinal strands (20). Snail-track degeneration, which was previously identified exclusively on clinical grounds, can now be clearly differentiated using SS-OCT based on its characteristic hyporeflective and granular reflective pattern (22).

SS-OCT also plays a crucial role in differentiating degenerative retinoschisis from rhegmatogenous retinal detachment, as it allows visualization of the typical bilaminar structure of retinoschisis, preservation of the outer retinal contour, and absence of subretinal fluid-findings that help prevent misdiagnosis and unnecessary intervention (23). This distinction is particularly important in patients with peripheral degenerative changes or high axial myopia.

Advances in UWF and SS-OCT technologies have also enabled a more detailed understanding of retinal tufts, which contemporary studies have identified as focal high-risk sites of abnormal vitreoretinal adhesion. UWF imaging facilitates their detection, while SS-OCT confirms the presence of unusually strong vitreoretinal connections and subclinical traction, particularly in young myopic individuals (24, 25). This is of particular clinical relevance in cases of acute PVD, where these focal adhesion sites often represent the initial locations of tractional retinal tear formation.

The increasing application of OCTA in peripheral retinal evaluation further enhances assessment of perfusion in atrophic and degenerative lesions. Recent studies suggest that OCTA can differentiate perfusion-deficient atrophic zones from areas with active vascular alterations, which may be useful in complex cases involving combined degenerative processes (24).

Overall, the contemporary diagnostic approach to peripheral retinal degenerations is based on the integration of indirect ophthalmoscopy, UWF imaging, and SS-OCT analysis, with each modality providing unique and complementary information. This multimodal strategy is now considered the emerging standard of clinical practice and allows more accurate risk stratification of individual peripheral lesions, directly informing decisions regarding observation versus prophylactic intervention (20–25).

### 2.4 What modern imaging has changed - and what it has not

The introduction of UWF imaging and swept-source OCT has fundamentally improved visualization of the retinal periphery and the vitreoretinal interface. These modalities have enabled detection of microstructural features such as focal vitreoretinal traction, retinal thinning, and bridging strands that were previously inaccessible to routine examination. Importantly, modern imaging has improved differentiation between degenerative retinoschisis and subclinical RRD, thereby reducing unnecessary prophylactic interventions.

However, increased diagnostic sensitivity does not equate to increased clinical risk. A substantial proportion of peripheral abnormalities detected on UWF and OCT remain clinically stable and do not require treatment. Indirect ophthalmoscopy with scleral depression remains indispensable for dynamic assessment of traction, and imaging findings must always be interpreted in conjunction with clinical symptoms and vitreous status. Thus, modern imaging should be viewed as a tool for risk stratification rather than a trigger for routine intervention.

### 2.5 Therapeutic dilemmas and prophylactic laser photocoagulation

The therapeutic management of peripheral retinal degenerations represents one of the most controversial areas in vitreoretinal medicine. Although many degenerative lesions are benign and stable, certain entities carry a clearly increased risk for the development of peripheral retinal tears or rhegmatogenous retinal detachment, naturally raising the question of patient selection for prophylactic laser photocoagulation (26, 27). Decision-making is further complicated by the fact that modern diagnostic modalities, including UWF imaging and SS-OCT, reveal a large number of peripheral abnormalities whose true clinical significance is not always well defined.

The central dilemma arises from the heterogeneity of available evidence regarding the effectiveness of prophylactic laser treatment. Earlier studies suggested that prophylactic photocoagulation may reduce the risk of retinal detachment in eyes with tractional degenerations or symptomatic retinal tears, whereas more recent literature indicates that many high-risk lesions may remain stable even in the absence of intervention (26). Consequently, clinical decision-making is generally guided by lesion type, symptom status, vitreous status, refractive profile, and history of retinal detachment, although available recommendations remain heterogeneous and are not uniformly supported by high-level evidence. Because the available literature remains heterogeneous and does not provide uniform quantitative risk estimates for all PRD subtypes and clinical scenarios, the categories presented in **Tables 1**–**3** should be interpreted primarily as literature-informed qualitative risk strata. Quantitative estimates are included only where sufficiently consistent evidence is available.

**Table 1:** Pragmatic clinical framework for assessing the need for prophylactic laser photocoagulation in patients with symptomatic PVD.

Clinical scenario	Findings	Risk	Recommendation
Symptoms of acute PVD (photopsias, floaters)	Retinal tear present	High	Urgent prophylactic laser photocoagulation
Symptoms of acute PVD	Retinal tear with limited subretinal fluid (SRF)	Extremely high	Immediate laser photocoagulation (absolute indication)
Acute PVD	No retinal tear, but vitreoretinal traction on lattice degeneration/snail-track lesion/retinal tuft	Moderate to high	Consider laser photocoagulation based on symptoms and OCT findings
Acute PVD	No retinal tear and no vitreoretinal traction	Low (<2%)	No laser treatment; follow-up in 4–6 weeks

**Table 2:** Literature-informed risk stratification framework for asymptomatic peripheral retinal degenerations according to lesion type and vitreous status.

Lesion type	Vitreous status	Risk of retinal tear/RRD	Recommended management
Paving-stone atrophy	No PVD/complete PVD	Very low	Observation only; no prophylactic laser
White-without-pressure (WWOP)	Any vitreous status	Very low	Observation only; routine follow-up
Degenerative retinoschisis (stable)	No PVD	Low	Observation; no laser treatment
Degenerative retinoschisis	Partial or acute PVD	Low to moderate (only if both layers are broken)	Observation; laser only if full-thickness breaks are present
Lattice degeneration	No PVD	Low	Observation; patient education
Lattice degeneration	Partial or acute PVD without traction	Moderate	Individualized follow-up; no routine laser
Lattice degeneration	Partial or acute PVD with OCT-confirmed traction	High	Consider prophylactic laser photocoagulation
Snail-track degeneration	No PVD	Low	Observation
Snail-track degeneration	Partial or acute PVD with traction	High	Consider prophylactic laser photocoagulation
Retinal tufts (meridional/nonpigmented)	No PVD	Low	Observation
Retinal tufts	Acute PVD with traction	Moderate to high	Individualized decision; laser if traction is present

**Table 3:** Pragmatic preoperative framework for considering prophylactic laser photocoagulation of peripheral retinal degenerations in patients scheduled for cataract surgery, refractive procedures, or Nd: YAG capsulotomy.

Clinical scenario	Lesion type	Vitreous status	Recommendation
Preoperative examination – no PRD	—	—	No prophylaxis; no delay of surgery
Benign PRD (paving-stone atrophy, WWOP)	Benign	Any vitreous status	No prophylaxis; no delay of surgery
Degenerative retinoschisis	Benign	No PVD	No laser treatment
Lattice/snail-track degeneration	Tractional	Complete PVD	Generally no prophylactic laser
Lattice/snail-track degeneration	Tractional	Partial PVD	Individualized decision based on overall risk
Lattice/snail-track degeneration	Tractional	Anticipated surgery-induced PVD	Consider prophylactic laser 10–14 days prior to surgery
PRD with history of RRD in the fellow eye	High risk	Any vitreous status	Prophylactic laser recommended
Multiple PRD with high myopia	Tractional lesions	Any vitreous status	Prophylactic laser considered on a case-by-case basis

In the setting of acute, symptomatic PVD, clinical decisions are generally more straightforward (**Table 1**). The presence of a retinal tear-regardless of whether it is accompanied by limited subretinal fluid-constitutes an absolute indication for urgent laser photocoagulation. The same principle may apply to carefully selected tractional lesions identified during acute symptomatic PVD when clinical examination demonstrates a suspicious vitreoretinal interface and multimodal imaging supports the presence of focal traction. Such scenarios carry the highest risk for horseshoe tear formation and subsequent progression, and timely intervention significantly reduces the likelihood of rhegmatogenous retinal detachment (28).

In contrast to symptomatic cases, asymptomatic peripheral retinal degenerations require a far more nuanced approach (**Table 2**). Benign lesions such as paving-stone atrophy, white-without-pressure, and stable degenerative retinoschisis do not confer a risk for rhegmatogenous retinal detachment and therefore are not candidates for prophylactic treatment (26, 27).

Conversely, tractional degenerations such as lattice and snail-track lesions exhibit a highly variable risk profile depending on vitreous status. In the absence of PVD, these lesions generally remain clinically stable; however, partial or acute PVD may substantially increase the risk, particularly when subclinical traction is detected on OCT imaging. In such circumstances, the decision regarding prophylactic intervention must be individualized and based on an integrated assessment of multimodal imaging findings and relevant clinical factors (21, 26, 27, 29).

Retinal tufts represent a distinct entity. Although they are typically stable in the absence of PVD, their role as focal initiation sites for retinal tears during acute PVD renders them clinically relevant, especially in myopic patients and in individuals with a history of retinal detachment in the fellow eye (23, 25).

Additional complexity arises in preoperative settings, particularly in patients scheduled for cataract surgery, refractive procedures, or Nd: YAG capsulotomy (**Table 3**). In the past, routine prophylactic treatment of lattice degeneration prior to intraocular interventions was common practice; however, more recent literature and expert recommendations suggest that such an approach is generally not justified in the absence of clearly defined additional risk factors, given that prophylactic laser photocoagulation is not without potential complications and has not shown consistent benefit in low-risk asymptomatic cases (27).

Currently, prophylactic treatment is recommended only in selected situations, including eyes with partial PVD accompanied by traction, eyes with a history of rhegmatogenous retinal detachment in the fellow eye, cases of high myopia associated with multiple peripheral degenerations, or when OCT clearly demonstrates unstable vitreoretinal adhesion (28).

When all of these considerations are viewed together, it becomes evident that optimal management of peripheral retinal degenerations cannot be based on a routine, uniform approach but must instead be strictly individualized. Integration of the criteria outlined in **Tables 1**–**3** allows the benefits of prophylactic intervention to be maximized in situations where it is truly indicated, while simultaneously avoiding unnecessary treatment of stable, low-risk lesions. This multimodal, stratified approach is consistent with the current understanding of vitreoretinal biomechanics and represents the most effective strategy for the prevention of rhegmatogenous retinal detachment (26, 27).

### 2.6 AI-assisted risk stratification and decision support

Modern peripheral imaging has markedly increased detection of PRD, particularly with UWF and peripheral SS-OCT. At the same time, it has expanded the “gray zone” between what is visible and what is clinically actionable. Many PRD identified on imaging remain stable over long periods, yet their appearance can trigger variable interpretation and inconsistent management, ranging from overly conservative follow-up to unnecessary prophylactic laser. The core challenge is therefore not detection itself, but standardized risk interpretation, especially in scenarios where the risk of RT and RRD is time-dependent, such as acute symptomatic PVD.

We propose an AI-assisted, imaging-guided risk stratification framework that integrates standardized clinical variables with multimodal imaging features from UWF and SS-OCT, with the explicit purpose of decision support (30). Clinical inputs include symptom profile consistent with acute PVD (photopsias, new floaters, subjective VF defect), refractive and biometric risk (high myopia, increased AL), prior RRD in the fellow eye, and procedure-related risk that may accelerate PVD or alter vitreoretinal biomechanics (cataract surgery, Nd: YAG capsulotomy, refractive procedures, repeated IVI). These factors are combined with vitreous status and PVD stage (no PVD, partial PVD, acute symptomatic PVD, complete PVD), which remains the dominant biomechanical modifier of risk across PRD entities.

On the imaging side, the model relies on lesion-level features that reflect both morphology and stability. From UWF, relevant inputs include lesion type (lattice, snail-track, retinal tufts, degenerative retinoschisis, WWOP, paving-stone), lesion extent, quadrant distribution, proximity to the vitreous base, and co-existence of multiple tractional lesions. From SS-OCT, particular weight is assigned to signs of instability, including focal vitreoretinal adhesion at lesion margins, bridging strands, localized contour irregularity, outer retinal disruption at traction points, microdefects suspicious for early breaks, and any SRF. In degenerative retinoschisis, SS-OCT features are especially valuable for distinguishing true schisis architecture from shallow RRD and for identifying high-risk configurations, particularly combined inner and outer layer breaks that permit communication between the vitreous cavity and the subretinal space.

The output of this framework is not a stand-alone diagnosis, but a structured risk category with an actionable recommendation. A practical implementation could generate a standardized report summarizing key inputs and issuing a decision-support suggestion such as urgent evaluation when high-risk features are present, short-interval review when risk is intermediate or uncertain, routine follow-up for stable low-risk lesions, or consideration of prophylactic laser in selected eyes with documented tractional instability and meaningful clinical risk factors. In symptomatic PVD, the system would prioritize detection and escalation pathways, flagging high-risk patterns (e.g., lattice/snail-track with traction on SS-OCT, suspicious posterior margin changes, focal hemorrhage adjacent to a tractional lesion) to ensure targeted re-examination, including scleral depression and repeat imaging where appropriate.

A central principle is a human-in-the-loop model (31). AI functions as a triage and documentation assistant that improves consistency and completeness of peripheral assessment, but the clinician remains responsible for confirmation and final decision-making (30, 31). This is critical because peripheral image quality is variable, artifacts can mimic pathology, and dynamic information obtained with scleral depression cannot be fully replaced by static imaging. Human oversight also mitigates the clinical consequences of false positives that could drive overtreatment and false negatives that could delay recognition of RT in symptomatic cases (31).

In everyday practice, AI-assisted decision support offers three realistic benefits. First, it standardizes interpretation by translating heterogeneous clinical impressions into a reproducible feature set, reducing interobserver variability in PRD assessment (32, 33). Second, it supports restraint by identifying truly low-risk lesions (e.g., traction-free WWOP or paving-stone, stable retinoschisis without high-risk breaks) and reinforcing observation with appropriate patient counseling rather than routine prophylaxis. Third, it improves triage and safety in acute PVD by highlighting configurations most associated with horseshoe RT and early RRD, prompting timely follow-up, targeted imaging, or urgent laser when indicated.

For responsible clinical translation, the framework should be developed with attention to bias, calibration, and external validity. Training and validation datasets must include diverse refractive profiles, ages, and imaging devices, with standardized labeling and clinically meaningful outcomes such as RT detection, development of RRD, and need for intervention. Transparent reporting of model performance, uncertainty estimates, and failure modes is essential before integration into routine workflows (34). In this context, AI is best framed as a pragmatic tool to operationalize imaging-guided, risk-based management of PRD by improving triage, standardizing documentation, and helping balance timely prophylaxis against avoidance of unnecessary intervention (35).

## 3 Discussion

Understanding the clinical significance of PRD remains challenging despite major advances in peripheral imaging. Although UWF and SS-OCT have improved detection and documentation of peripheral lesions, they have also increased the frequency of incidental findings whose true risk of progression is low or uncertain (25, 36). The central clinical task is therefore risk interpretation rather than lesion detection, with emphasis on identifying the minority of lesions that are biomechanically unstable and clinically actionable.

PVD remains the dominant pathophysiological event linking PRD to RT and subsequent RRD. During acute or incomplete PVD, traction is concentrated at focal points of strong vitreoretinal adhesion, most commonly at the posterior margins of lattice and snail track degeneration, where horseshoe RT frequently originate (20, 23). In clinical practice, the temporal association between acute PVD symptoms and localized tractional changes often carries greater decision making value than lesion morphology alone. SS-OCT has refined this concept by enabling *in vivo* visualization of microtraction, vitreoretinal strands, bridging structures, and focal outer retinal disruption, thereby improving biomechanical risk stratification beyond conventional ophthalmoscopy (20, 22).

A key implication of modern imaging is that increased detection does not automatically translate into increased treatment. The clearest evidence based indication for prophylactic laser remains symptomatic RT, particularly when accompanied by limited SRF, where the risk of progression is clinically meaningful and timely laser reduces the likelihood of RRD (26, 27, 29). In contrast, asymptomatic tractional lesions, including lattice and snail track degeneration, often remain stable in the absence of active traction or evolving PVD, and the balance of benefit versus harm generally favors observation and patient education (26, 27). From a practical perspective, appropriate restraint is as important as timely intervention, because unnecessary laser may increase focal adhesion, induce new breaks at treatment borders, or trigger exudative responses (26, 29).

Retinal tufts provide a useful example of why a purely morphology based approach is insufficient. They represent focal sites of abnormal vitreoretinal adhesion and can serve as initiation points for RT during acute PVD, yet most remain clinically quiescent in asymptomatic eyes (23, 25). Their relevance therefore depends on context, particularly symptoms, PVD stage, refractive profile, and imaging evidence of traction. Similarly, SS-OCT has materially improved differentiation between degenerative retinoschisis and subclinical RRD, reducing misclassification and unnecessary intervention. This remains clinically critical, because management differs substantially and prophylactic laser is not indicated in stable retinoschisis without high risk full thickness configurations (22).

Preoperative evaluation of the peripheral retina remains one of the most common real world dilemmas. Historically, routine prophylactic treatment of predisposing lesions was often performed before cataract surgery, refractive procedures, or Nd: YAG capsulotomy. Contemporary evidence does not support this approach in the absence of tractional instability or major risk modifiers such as a history of fellow eye RRD (29). In this setting, decision making should prioritize dynamic vitreoretinal status rather than static lesion classification alone. This is important because iatrogenic or surgery associated changes in the vitreous can shift risk in susceptible eyes, but indiscriminate prophylaxis exposes a large low risk population to procedure related harms without proven benefit (26–28).

Beyond imaging and intervention, structured patient education remains a cornerstone of RRD prevention. Prompt evaluation of warning symptoms, including photopsias, sudden increase in floaters, or a new peripheral VF defect, often has greater population level impact than widespread prophylactic treatment of stable lesions (27, 28). Clear counseling, written symptom instructions, and appropriate follow up intervals are therefore essential components of a risk based approach. To operationalize imaging-guided, risk-based management of PRD, we outline a conceptual AI-assisted decision-support framework integrating standardized clinical variables with UWF and peripheral SS-OCT features (**Table 4**).

**Table 4:** AI-assisted, imaging-guided risk stratification for PRD.

Component	Key inputs	Decision-support output
Clinical risk	Acute PVD symptoms; high myopia/axial length; fellow-eye RRD; procedure-related risk (cataract, Nd: YAG, refractive surgery, repeated IVI)	Risk category (low/intermediate/high) + suggested follow-up urgency
PVD status	No PVD/partial PVD/acute symptomatic PVD/complete PVD	Biomechanical risk modifier guiding escalation
UWF features	Lesion type and extent; quadrant distribution; proximity to vitreous base; multiple tractional lesions	Structured lesion map supporting consistent interpretation
Peripheral SS-OCT features	Signs of instability (focal adhesion, bridging strands, contour irregularity, microdefects, SRF)	“Instability” flag prompting targeted re-examination ± consideration of prophylactic laser in selected high-risk eyes
Human-in-the-loop	Image quality/artifact checks; clinician confirmation	AI supports triage and documentation; final decisions remain clinician-led

Finally, future progress is likely to depend on standardized risk stratification supported by multimodal imaging and, potentially, AI-assisted decision support. A human in the loop framework that integrates symptoms, refractive and surgical risk modifiers, PVD stage, and SS-OCT signs of tractional instability could reduce interobserver variability and help target prophylactic laser to eyes most likely to benefit, while minimizing overtreatment of benign PRD. Prospective studies using standardized definitions, imaging protocols, and clinically meaningful outcomes are needed to validate such approaches and refine thresholds for intervention (26–28, 37).

## 4 Conclusion

PRD comprise a heterogeneous spectrum of peripheral retinal changes in which clinical relevance is defined by the interaction between lesion morphology, vitreous status, and patient-specific risk factors. Contemporary imaging modalities, particularly UWF imaging and SS-OCT, have improved visualization of the retinal periphery and refined differentiation between low-risk and high-risk configurations by revealing microstructural signs of vitreoretinal traction, early RT formation, and subtle SRF. However, enhanced detection should not be equated with higher clinical risk, since the majority of benign PRD remain stable and do not require treatment. Prophylactic laser photocoagulation remains an effective preventive strategy, but its benefit is confined to carefully selected situations. Clear indications include symptomatic RT, RT with limited SRF, and acute symptomatic PVD in the presence of tractional instability documented clinically and or on SS-OCT, particularly in eyes with prior fellow-eye RRD or other major risk modifiers such as high myopia.

Conversely, routine prophylaxis is not justified in stable asymptomatic lesions, including paving-stone atrophy, WWOP, and traction-free lattice or snail-track degeneration, where observation and patient education represent the safest approach. Future progress in PRD management will increasingly rely on standardized, imaging-guided risk stratification and decision support, potentially supported by AI in a human-in-the-loop framework. Integrating clinical assessment with multimodal imaging and selective intervention provides the most balanced strategy to prevent RRD while minimizing unnecessary procedures and preserving patient safety. In current practice, the most appropriate management of PRD is not defined by lesion detection alone, but by careful integration of symptoms, vitreous status, multimodal imaging, and individualized clinical risk.
